# Evaluation of healthcare-related factors influencing mental health of Taiwanese citizens among different age groups

**DOI:** 10.1038/s41598-024-57675-x

**Published:** 2024-03-26

**Authors:** Yun-Hsiang Tien, Jingchi Huang

**Affiliations:** School of Political Science and Public Administration, Wuhan University, Wuhan, 430072 China

**Keywords:** Psychology, Health care, Health occupations

## Abstract

The issue of mental health has gained heightened recognition as a significant public health concern due to its potential to significantly impact various aspects of individuals’ lives. Numerous factors may influence mental health, and this study seeks to investigate and compare potential healthcare-related factors that affect the mental health of Taiwanese individuals across different age groups. Data for this study were taken from the Taiwan Social Change Survey (TSCS), conducted in 2021. Descriptive statistics were calculated to compare the three age groups. Then, multiple regression models were constructed with mental health conditions as the dependent variable and demographics and other key healthcare-related components as independent variables, respectively. Results showed that, among the three age groups, the middle-aged adults had the highest BMI, and the older adults had significantly better mental health. As compared with the other age groups, the older adults had significantly better perceptions of fair distribution of healthcare resources, and their trust in the healthcare system was the highest. With regard to searching for online healthcare information, the frequency reported by the older adults was the lowest. The regression model showed that, religious belief, trust in the healthcare system and searching for online healthcare information were significantly associated with mental health of middle-aged adults. In the younger group, searching for online healthcare information was significantly negatively associated with mental health. The study’s findings provide insight into how to provide Taiwanese citizens of different age groups with proper and targeted mental health promotion activities.

## Introduction

Since the end of 2019, the COVID-19 pandemic has become a major public health issue across the world. According to statistics released by the World Health Organization (WHO), worldwide deaths from COVID-19 surpassed 6 million by 2022^1^. In fact, severe pandemics such as COVID-19 affect all segments of the global population. Suffering from such a human, economic, and social crisis, both people’s physical and mental health have been unavoidably affected and cannot be neglected. In academia, since the initial outbreak of COVID-19, there have been a growing number of publications focusing on the impacts of the COVID-19 pandemic on mental health^2–5^. A large portion of these studies have examined symptoms and coping strategies for mental health issues such as anxiety and depression etc^6–8^. Others have analyzed the factors affecting people’s mental health during the COVID-19 pandemic^9–11^. The factors proposed in these empirical studies were diverse and cover different research objects such as students, patients, and medical caregivers, etc^12–14^. In 2020, Taiwan has been credited as one of the most successful areas in battling COVID-19, and at that time, the mental health impact of COVID-19 has been less prominent in Taiwan^15–17^. However, an outbreak in late April 2021 led to a sharp surge in cases^18^. What was the mental health status of of individuals during the widespread outbreak? Since 1995, a national health insurance (NHI) program has been in operation in Taiwan, which provides uniform comprehensive coverage. Taiwan’s healthcare system has been considered excellent in the world^19–21^. What healthcare-related factors influenced the mental health status of Taiwanese citizens? The objective of this study was to investigate the impact of healthcare-related factors on mental health and to assess potential variations in these factors among different age groups.

## Literature review and hypothesis

The inequitable allocation of healthcare resources is a global problem, as the equitable allocation of healthcare resources is one of the basic conditions for achieving healthcare fairness in the population^22^. During pandemics such as COVID-19, issues around healthcare resources fairness draw substantial public attention and discussion^23–25^. Although it is generally believed that limited medical resources should be allocated to do the greatest good for the greatest number of people, many questions about what is “fair” still remains unanswered^26^. One panel of experts argued that pregnant mothers, mothers of children under 5 years, age groups younger than 80, and front-line healthcare workers should have high priority in allocating mechanical ventilators^27^. Some researchers proposed to give vulnerable populations higher priority in the distribution of medical resources^28^. Past studies have showed that fairness in treatment is a predictor of satisfaction with care, and public satisfaction with healthcare and perceived fairness in healthcare are positively correlated^29,30^. Therefore, perceptions of the fair distribution of healthcare resources could be a factor in contributing to good mental health during the COVID-19 pandemic. In this regard, we propose the following research hypothesis:

### Hypothesis 1: The more individuals feel that healthcare resources are fairly distributed, the better their mental health is.

People’s trust in the healthcare system plays a role in explaining their access to and utilization of medical care, adherence to medications, continuity of care, and even their self-reported health status^31^. An absence of trust might have harmful effects on the health of patients, as patients may refuse to seek doctors’ professional assistance or withhold information about their illness conditions^32^. Hence, trust in the healthcare system has been considered to be a factor affecting mental health status; in particular, during the COVID-19 pandemic, healthcare system mistrust has been associated with an increased likelihood of psychological distress^33^. In this regard, we propose the following research hypothesis:

### Hypothesis 2: The stronger trust individuals have in the healthcare system, the better their mental health is.

People tend to use the internet to find healthcare information, and online information is available on diverse topics such as specific diseases, illnesses, advice on diet and nutrition, etc^34^. A prior study has indicated that there is a statistically significant relationship between a diagnosis of depression and anxiety disorder (DAD) and the use of online social media^35^. On the one hand, online healthcare information search behavior can help people effectively understand their health status and thereby reduce their anxiety and other negative emotions^36^. On the other hand, during public health crises such as the COVID-19 pandemic, excessive or repeated searches for online healthcare information may increase perceptions of threat and uncertainty^37^. As a result, the influence of online healthcare information search behavior on the mental health needs further testing. In this regard, we propose the following research hypothesis:

### Hypothesis 3: The more frequently individuals search for online healthcare information, the worse their mental health is.

In addition to analyzing people’s mental health conditions and related influencing factors of people, it is important to compare these factors among different age groups. Age (or generation) has been one of the demographic variables that has received substantial interest in mental health research^38,39^. Some studies focused on a specific age group for analysis, while others compared different age groups^40–43^. Many studies have paid attention to different age groups influenced by the COVID-19 pandemic^44–46^. It is likely that the effects caused by pandemics are experienced differently among age groups^47^. To confirm whether mental health and healthcare-related influencing factors have differed among age groups during the COVID-19 pandemic, the present study used to a dataset from a representative sample collected via an academic project executed by a credible academic institution. The importance of mental health cannot be neglected because individuals with mental illness usually experience poorer physical health in comparison to the general population^48^. Therefore, the present study aimed to compare mental health status, perceptions of the fair distribution of healthcare resources, trust in the healthcare system, and searching for online healthcare information among different age groups of Taiwanese citizens. In addition, through statistical analysis, this study tested the correlations between the aforementioned variables in each of the different age groups separately. The findings thereby obtained make it possible to arouse the attention of the public, and help the healthcare and public health sector to understand the views and behaviors of different age groups, and then propose appropriate policies towards then respectively.

## Methods

### Participants

The dataset used in the present study was collected via a research project in Taiwan (i.e. the Taiwan Social Change Survey [TSCS]). The target population in the TSCS was residents of Taiwan (i.e. those recorded in the Department of Household Registration), and the TSCS adopted a stratified random sampling method. Data collection for the TSCS was arranged and completely supervised by the Institute of Sociology, Academia Sinica (Taiwan)^49^. For the 2021 TSCS, in-person interviews were conducted between September 2021 and February 2022. The institutional review board (IRB) for Humanities & Social Science Research Academia Sinica (AS-IRB-HS 02-19034[R5]) approved the TSCS data collection. In addition, participants involved in data collection for the TSCS were 18 years old and above and provided informed consent for participation. The survey was conducted by in-person interviews. The 2021 TSCS data collection period overlapped with the worldwide COVID-19 pandemic, and the pandemic made it difficult to conduct face-to-face data collection. The 2021 TSCS had less respondents than the TSCS executed before 2020, with a final sample size of 1604 participants.

### Measures

#### Mental health conditions

Five items, rated using a five-point Likert scale from 1 = “always” to 5 = “never”, were used to measure mental health status. The five items were “How often do you feel unhappy or depressed?”, “How often do you lose confidence in yourself?”, “How often do you feel unable to overcome the difficulties you face?”, “How often do you feel moody and blue?”, and “How often do feel calm and at peace?”. All item scores were coded such that a higher score indicated better mental health status. The KMO (Kaiser–Meyer–Olkin) coefficient of the five items was 0.87, and this result showed that the instrument met the validity criteria. In addition, Cronbach’s alpha indicated good internal consistency for this scale in the current study (α = 0.88). The item scores were then averaged to calculate an overall mental health status score.

#### Perceptions of fair distribution of healthcare resources

Four items were rated using a five-point Likert scale from 1 = “much easier” to 5 = “much more difficult” to assess attitudes about the fair distribution of healthcare resources for four kinds of population (i.e. rich vs. poor, elderly vs. young, female vs. male, and citizens vs. non-citizens). In the descriptive statistics and multiple regression analysis, 1 meant “much easier” or “much more difficult”, 2 meant “easier” or “difficult”, and 3 meant “no difference”. A higher score indicated a higher level of fair distribution of healthcare resources. The KMO coefficient of the four items was 0.62, and this result showed that the instrument met the validity criteria. In addition, Cronbach’s alpha indicated acceptable internal consistency for this scale in the current study (α = 0.54). The items scores then averaged to calculate an overall score in perceptions of fair distribution of healthcare resources.

#### Trust in the healthcare system

Five items were used to assess individuals’ trust in the healthcare system. They were rated using a five-point Likert scale from 1 = “totally distrust/disagree” to 5 = “totally trust/agree”. The five items were “In the next few years, Taiwan’s healthcare system will become better.”, “On the whole, Taiwan’s healthcare system is efficient.”, “Do you think you can get the best treatment when you are seriously ill?, “On the whole, doctors can be trusted.”, and “Generally speaking, do you trust Taiwan’s healthcare system?”. All item scores were coded such that a higher score indicated a higher level of trust in the healthcare system. The KMO coefficient of the five items was 0.72, and this result showed that the instrument met the validity criteria. In addition, Cronbach’s alpha indicated acceptable internal consistency for this scale in the current study (α = 0.62). The item scores were then averaged to calculate an overall score in trust in the healthcare system.

#### Searching for online healthcare information

Three items were rated using a five-point Likert scale from 1 = “never” to 5 = “always” to assess the different kinds of healthcare information that the respondents searched for on line (i.e. healthy lifestyle, questions about anxiety and stress, and vaccinations). A higher score indicated a higher frequency of searching for online healthcare information for that topic. The KMO coefficient of the three items was 0.64, and this result showed that the instrument met the validity criteria. In addition, Cronbach’s alpha indicated acceptable internal consistency for this scale in the current study (α = 0.70). The item scores were then averaged to calculate an overall a score in frequency of searching for online healthcare information.

#### Age groups

In the survey, the respondents were asked their birth year; the present study divided them into three age groups. With reference to the classification methods used by many researchers in the past, this study categorized participants by age into younger adults (ages 18–35 years), middle-aged adults (ages 36–55 years), and older adults (aged older than 55 years)^50^.

#### Demographic variables

In addition to information about their age, the participants were also asked various questions concerning other demographic information: gender (male or female); height (in centimeters); weight (in kilograms); schooling years (since elementary school); relationship status (single or non-single) ( To simplify the response choices and then facilitate the statistical analysis, single comprised “unmarried”, “separated”, “divorced”, and “widowed”, while non-single comprised “first marriage”, “remarried”, and “cohabitation”.); religious belief (yes or no); residency (big urban areas and others); employment status (full-time employment and others); household monthly income. Height and weight were used to calculate body mass index (BMI). In terms of the household monthly income, the initial questionnaire included 26 choices, spanning from zero income to over NT$ 1 million.

### Statistical analyses

All data were summarized using descriptive statistics (including means, standard deviations, frequencies, and percentages) to portray the features of the studied variables in the TSCS 2021. ANOVA tests (for continuous data) and χ^2^ tests (for categorical data) were used to examine the differences among the three age groups. Three multiple regression models were then constructed for the three age groups using parallel variables: the dependent variable was mental health condition; the independent variables were perceptions of the fair distribution of healthcare resources, trust in the healthcare system, searching for online healthcare information, other demographic variables excluding age. All the statistical analyses were executed through SPSS (Statistical Product Service Solutions) 27.0.

### Institutional review board statement

The institutional review board (IRB) for Humanities & Social Science Research Academia Sinica (AS-IRB-HS 02-19034 [R5]) approved the TSCS data collection. All methods were performed in accordance with the Declaration of Helsinki and participants could withdraw consent at any time. Also, the participants’ right to data confidentiality is considered and protected in the TSCS data collection, which adheres to the compliance with the Declaration of Helsinki.

### Compliance statement

All methods were carried out in accordance with relevant guidelines and regulations.

## Results

### Demographic comparisons among different age groups

The respondents’ demographic information for each of the three age groups is presented on Table 1. No significant differences were found in the gender (p = 0.165) and residency (p = 0.347) distributions. Among the three age groups, significant differences were found in the average of BMI (24.07 vs 24.61 vs 23.87, p = 0.007), schooling years (9.94 vs 13.05 vs 14.83, p < 0.001), household monthly income (5.90 vs 9.49 vs 10.65, p < 0.001), relationship status (37.9% vs 33.2% vs 58.6% for single, 62.1% vs 66.8% vs 41.4% for non-single; p < 0.001), religious belief (85.8% vs 76.8% vs 57.0% for yes, 14.2% vs 23.2% vs 43.0% for no; p < 0.001), and employment status (20.2% vs 67.4% vs 75.6% for full-time, 79.8% vs 32.6% vs 24.4%; p < 0.001).

**Table 1:** Demographic comparisons among different age groups.

	Older adults (n = 446)	Middle-aged adults (n = 626)	Younger adults (n = 532)	p-value
	M (SD) or n (%)	M (SD) or n (%)	M (SD) or n (%)	
Gender				
Males	200 (44.8)	287 (45.8)	268 (50.4)	0.165
Females	246 (55.2)	339 (54.2)	264 (49.6)	
Schooling years	9.94 (4.16)	13.05 (3.73)	14.83 (2.51)	< .001
Relationship status				
Single	169 (37.9)	208 (33.2)	312 (58.6)	< .001
Non-single	277 (62.1)	418 (66.8)	220 (41.4)	
BMI (kg/m^2^ )	24.07 (3.54)	24.61 (4.00)	23.87 (4.58)	0.007
Religious belief				
Yes	382 (85.8)	481 (76.8)	303 (57.0)	< .001
No	63 (14.2)	145 (23.2)	229 (43.0)	
Residency				
Big urban areas	111 (24.9)	155 (24.8)	150 (28.2)	0.347
Others	335 (75.1)	471 (75.2)	382 (71.8)	
Employment status				
Full-time	90 (20.2)	422 (67.4)	402 (75.6)	< .001
Others	356 (79.8)	204 (32.6)	130 (24.4)	
Household monthly income	5.90 (4.35)	9.49 (5.49)	10.65 (5.49)	< .001

### Mental health conditions and healthcare-related factors among different age groups

Table 2 shows the differences in mental health conditions, perceptions of the fair distribution of healthcare resources, trust in the healthcare system, and searching for online healthcare information among the three age groups. The older group had significantly better mental health conditions (4.32 vs 4.08 vs 3.92; p < 0.001) , perceptions of the fair distribution of healthcare resources (2.23 vs 2.14 vs 2.14; p = 0.012) and trust in the healthcare system (3.89 vs 3.74 vs 3.81; p < 0.001) than the other age groups. With regard to searching for online healthcare information, the frequency reported by the older group was the lowest, and that reported by the younger group was the highest (1.95 vs 2.35 vs 2.61; p < 0.001).

**Table 2:** Mental health conditions and healthcare-related factors among different age groups.

	Older adults (n = 446)	Middle-aged adults (n = 626)	Younger adults (n = 532)	p-value
	M (SD)	M (SD)	M (SD)	
Mental health conditions	4.32 (0.82)	4.08 (0.75)	3.92 (0.73)	< .001
Perceptions of the fair distribution of healthcare resources	2.23 (0.48)	2.14 (0.45)	2.14 (0.43)	0.012
Trust in the healthcare system	3.89 (0.50)	3.74 (0.57)	3.81 (0.51)	< .001
Searching for online healthcare information	1.95 (0.91)	2.35 (0.85)	2.61 (0.69)	< .001

### Multiple linear regression model explaining mental health among age groups

Table 3 presents the results of the multiple regression models. In the older group, the regression equations were not significant in model 1 and model 2. In the middle-aged group, in both Model 3 and model 4, the regression equations showed statistical significance. In Model 3, individuals who did not have no religious belief had worse mental health than those who had (β = − 0.148; p < 0.001). When the healthcare-related factors were added in the equation, in Model 4, in addition to the significant influence of religious beliefs on individuals’ mental health (β = − 0.152; p < 0.001), individuals with higher levels of trust in the healthcare system had better mental health than those with lower levels of trust (β = 0.195; p < 0.001); individuals who spent more time searching for online healthcare information had worse mental health than those who spent less (β = − 0.170; p < 0.001). In the younger group, the regression equation was not significant in model 5. When the healthcare-related factors were added in the equation, in Model 6, the equation was significant. Individuals who spent more time searching for online healthcare information had worse mental health than those who spent less (β = − 0.170; p < 0.001).

**Table 3:** Multiple linear regression model explaining mental health of different age groups.

	Older adults (n = 446)		Middle-aged adults (n = 626)		Younger adults (n = 532)	
	Model 1	Model 2	Model 3	Model 4	Model 5	Model 6
	Beta (t)	Beta (t)	Beta (t)	Beta (t)	Beta (t)	Beta (t)
Gender (Ref: male)	− 0.121 (− 1.486)	− 0.132 (− 1.618)	− 0.072 (− 1.480)	− 0.052 (− 1.097)	− 0.131* (− 2.422)	− 0.102 (− 1.894)
Schooling years	0.006 (0.070)	0.086 (0.942)	− 0.046 (− 0.902)	− 0.009 (− 0.167)	0.051 (0.989)	0.088 (1.716)
Relationship status (Ref: single)	0.081 (0.994)	0.074 (.915)	0.059 (1.225)	0.072 (1.534)	0.044 (0.851)	0.045 (0.881)
BMI	0.047 (0.608)	0.043 (0.566)	0.068 (1.449)	0.061 (1.335)	0.014 (0.267)	0.013 (0.249)
Religious belief (Ref: yes)	− 0.086 (− 1.055)	− 0.074 (− 0.919)	− 0.148*** (− 3.229)	− 0.152*** (− 3.386)	0.025 (0.509)	0.012 (0.240)
Residency (Ref: Big urban areas)	− 0.094 (− 1.168)	− 0.086 (− 1.053)	0.034 (0.731)	0.032 (0.685)	0.024 (0.492)	0.022 (0.443)
Employment status (Ref: full-time)	0.160* (2.079)	0.139 (1.806)	− 0.021 (− 0.441)	− 0.013 (− 0.272)	− 0.029 (− 0.569)	− 0.040 (− 0.790)
Household monthly income	0.061 (0.699)	0.021 (0.237)	0.101 (1.836)	0.084 (1.559)	0.047 (0.870)	0.037 (0.687)
Perceptions of the fair distribution of health resources		0.002 (0.026)		− 0.038 (− 0.854)		− 0.019 − 0.395)
Trust in the healthcare system		0.143 (1.827)		0.195*** (4.287)		0.093 (1.927)
Searching for online health information		− 0.141 (− 1.646)		− 0.170*** (− 3.578)		− 0.170*** (− 3.408)
F	1.441	1.725	3.321**	5.224***	1.709	2.635**
R^2^	0.065	0.104	0.055	0.112	0.032	0.066
∆F	1.441	2.384	3.321**	9.791***	1.709	4.973**
∆R^2^	0.065	0.039	0.055	0.057	0.032	0.034

***p < 0.001, **p < 0.01, *p < 0.05.

## Discussion

Through statistical analysis of the survey data, the present study discovered differences in the mental health and healthcare-related factors among Taiwanese citizens of different age groups. In all three age groups, the perceptions of the fair distribution of healthcare resources did not have significant associations with the mental health condition. Therefore, the hypothesis 1 was not supported. The trust in the healthcare system had significantly positive associations with the mental health condition only in the middle-aged group. Consequently, the hypothesis 2 was partially supported. Searching for online health information had significantly negative associations with the mental health condition only in the middle-aged and younger groups. As a result, the hypothesis 3 was partially supported.

With regard to mental health, the older adults appeared to be happier than the other age groups. Unlike their successors, the older adults had gradually gone into retirement, and for the most part, their children had grown into adulthood. In other words, their burdens of family and work had gradually lessened, and thus they tended to experience “the age-related positivity effect”, an increasing focus on positive events and happy feelings^51^. In addition, generally speaking, individuals of the middle-aged and younger groups are likely to encounter work-family conflict causing fear, stress, loneliness, panic, depression, fear of worthlessness, etc^52,53^. It is important to give them sufficient social support, and they also have to actively ask for help. In Taiwan, there are several public and non-profit organizations that aim to serve people who encounter psychological problems, and citizens should clearly understand how to access these services when in need.

With regard to the perceptions of the fair distribution of healthcare resources, the older adults felt more strongly than the other age groups that healthcare resources were fairly distributed. These findings reflect a certain amount of inter-generational antagonism in Taiwan^54^. In the recent decade, Taiwan’s stagnant wages have become a barrier for the younger and middle-aged adults in getting married, raising a family, and buying a house. The generational wealth gap is worsening, fueling discontent among younger people^55^. Facing a more difficult and rigorous environment than the time of their predecessors, the younger and middle-aged people are likely to have a deeper feelings about the gap between the rich and the poor and between the elderly and the young.

The middle-aged adults had lower trust in the healthcare system than the other age groups, and their trust in the healthcare system was positively associated with the mental health condition. Middle-aged adults may have a heightened awareness of healthcare service due to their responsibilities in caring for both older family members and children. These findings indicate that middle-aged people have greater demands and standards for the quality of healthcare services. In this regard, governments and hospitals should make efforts to enhancing healthcare services and boosting citizens’ satisfaction with medical care.

For the middle-aged and younger groups, searching online for healthcare information had significantly negative associations with the mental health condition. These findings echo evidence from prior studies showing that individuals’ low levels of self-rated health and high levels of perceived psychological distress make search for healthcare-related information via the Internet in order to cope with their healthcare-related concern and distress^56,57^. In modern society, due to privacy concerns, when encountering psychological problems, some people may tended to resort to seeking online help prior to face-to-face diagnosis^58^. Furthermore, the COVID-19 pandemic has reduced the intention and the frequency of citizens to go to the hospital because of fear of becoming infected^59^. Consequently, whether during a pandemic or not, governments and hospitals should make efforts to provide the public with complete and up-to-date information about psychological problems. Moreover, all types of mass media should also help spread information so that people in need can obtain useful help in time.

Finally, according to the results of this study, religious belief had a positive impact on mental health for the middle-aged adults. Many studies have examined the link between religious belief, religious practice, and mental health, and have found that higher levels of religious belief and practice are associated with better mental health^60,61^. For this reason, choosing a suitable religious belief might be a feasible to release distress in daily life.

The present study has some limitations that should be discussed. First, given that the study used secondary data analysis, the items comprising each concept could not be designed or modified, with the result that some important variables were not fully assessed in the survey. Second, all the survey items were completed through self-reports, and therefore common method bias was inevitable. Third, there is more than one way to determine when each age begins and ends, and different ways of dividing the age groups might cause slight differences in the research results.

## Conclusion

The present study showed that there are age differences in healthcare-related factors influencing the mental health of Taiwanese citizens. More specifically, by comparing the three age groups, we found that (i) the older adults had better mental health, (ii) the older adults had better perceptions of the fair distribution of healthcare resources, (iii) the older adults had higher trust in the healthcare system, (iv) the younger adults searched for online health information more frequently. Moreover, religious belief was significantly associated with mental health in the middle-aged group. Consequently, to improve the mental health of citizens, it is important for the Taiwanese government and hospitals to provide enough online resources about psychological assistance for the public as much as possible.

## Data Availability

The information of data and materials could be obtained from the TSCS website, https://srda.sinica.edu.tw.
